# An aberrant mediastinal medial basal segmental pulmonary artery (A7a) in a patient with lung cancer: a case report

**DOI:** 10.1186/s40792-021-01112-y

**Published:** 2021-01-13

**Authors:** Yoshiaki Takase, Hiroyoshi Tsubochi, Ei Yamaki, Osamu Kawashima

**Affiliations:** 1 Department of Thoracic Surgery, Shibukawa Medical Center, 383 Shiroi, Shibukawa, Gunma 377-0280 Japan; 2 Department of Thoracic Surgery, Jichi Medical University, 3311-1 Yakushiji, Shimotsuke, Tochigi 329-0498 Japan

**Keywords:** Lung cancer, Pulmonary artery abnormality, Segmentectomy

## Abstract

**Background:**

Mediastinal branching of the A7a from the right main pulmonary artery (PA) is extremely rare. Herein, we report a patient with an aberrant mediastinal A7a who underwent right basal segmentectomy for lung cancer.

**Case presentation:**

A 73-year-old man was referred to our department for a right lower lobe nodule measuring 18 mm in diameter on computed tomography (CT). Three-dimensional (3D) CT revealed mediastinal A7a branching from the right main PA. As the patient had undergone colectomy for advanced ascending colon cancer, the nodule was suspected to be a metastasis from the colon primary, and thus, basal segmentectomy of the right lung was performed. Intraoperatively, the A7a was observed behind the V4+5 and middle lobe bronchus. The pathological diagnosis was combined small cell carcinoma with an adenocarcinoma component (p-T1cN0M0, stage IA3). The patient subsequently received adjuvant chemotherapy for colon cancer. At 1-year postoperative follow-up, there was no evidence of disease.

**Conclusion:**

This is the first report describing an aberrant mediastinal A7a branching from the right main PA. It is important to obtain accurate information about variations of the PA using 3D-CT for safe anatomical pulmonary resection.

## Background

Only a few published reports have documented a lung resection with an anatomical abnormality of A7 [1, 2]. To the best of our knowledge, there are no reports that demonstrate an isolated anatomical abnormality of the origin of A7a from the right main pulmonary artery (PA), while the A7b branches as usual, in a patient undergoing lung resection. Herein, we report a rare case with an aberrant branch of the right medial basal segmental PA (A7a) from the right main PA.

## Case presentation

**Fig. 1:**
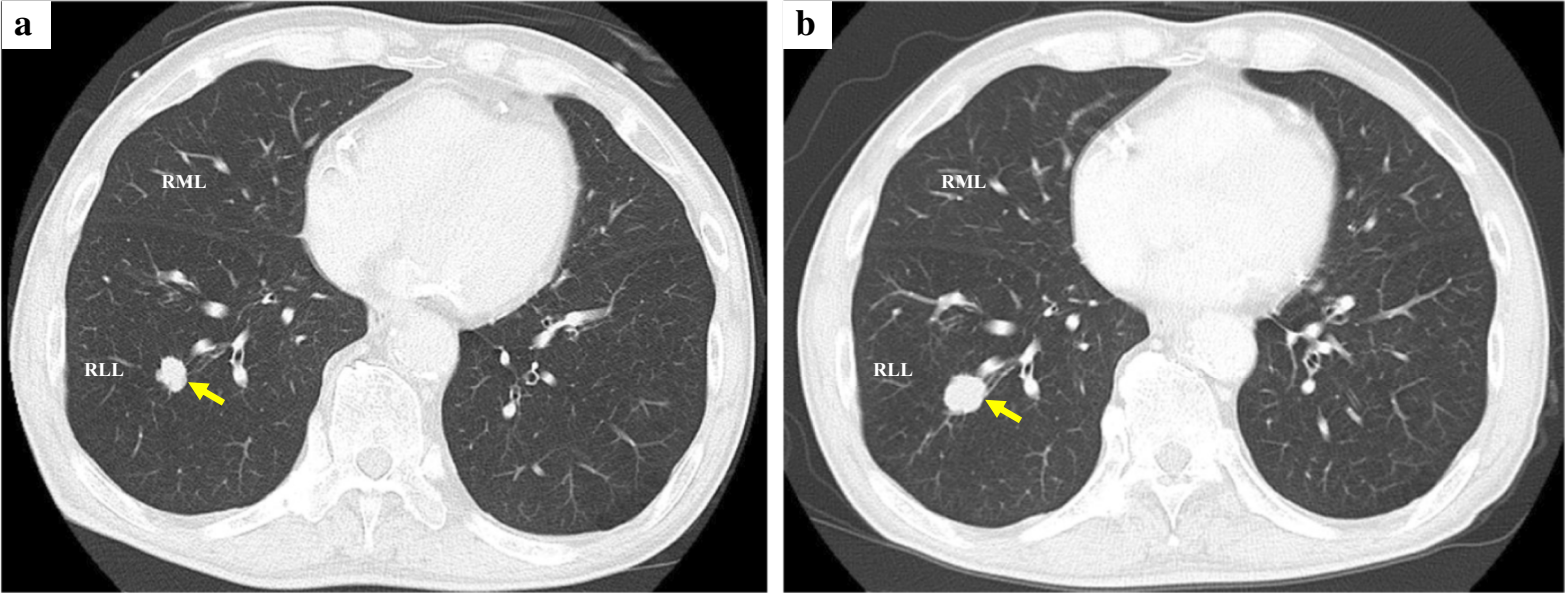
Axial section of computed tomography findings. The tumor is located in S9 of the right lung (arrow). **a** Four months before lung resection. **b** One month before lung resection. *RLL* right lower lobe; *RML* right middle lobe

**Fig. 2:**
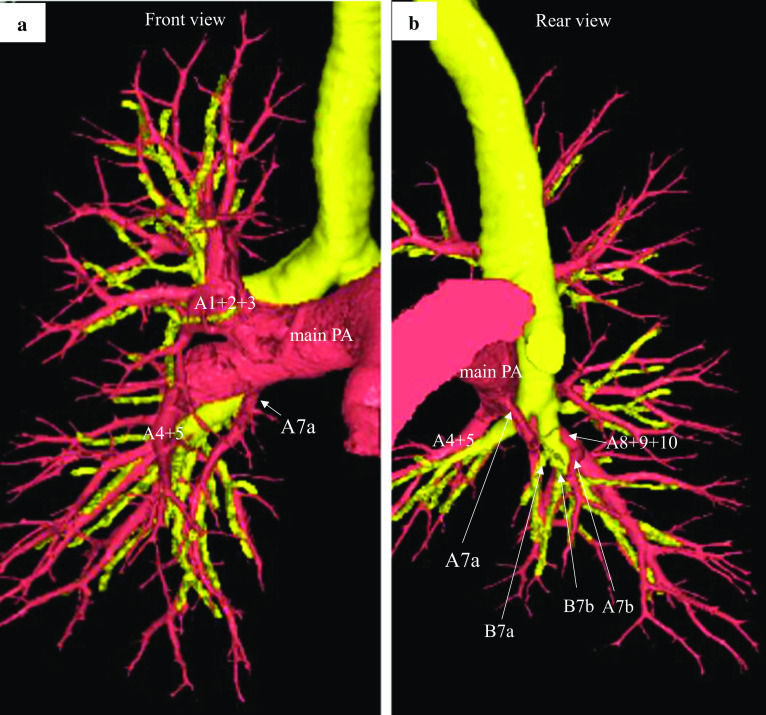
Three-dimensional computed tomography angiography and bronchography images. **a** Front view. **b** Rear view. Reconstructed images of arteries (red), veins (blue), bronchi (yellow), and tumor (green). *A* artery; *B* bronchus; *V* vein

A 73-year-old man was diagnosed with colon cancer and underwent right hemicolectomy at our hospital. The final diagnosis of colon cancer revealed adenocarcinoma, type 2, 70 × 40 mm, tub2 > tub1, T4a (SE), int, INFb, Ly1a, V1b, Pn0, N1a (1/30), p-stage IIIB (according to the ninth edition of TNM classification). Computed tomography (CT) before surgery for colon cancer revealed a solid nodule measuring 16 mm in diameter in the laterobasal segment (S9) of the right lung (Fig. 1a). Three months after the surgery for colon cancer, the size of the pulmonary nodule increased (diameter, 18 mm; Fig. 1b). The levels of tumor markers, including carcinoembryonic antigen (1.7 ng/mL), carbohydrate antigen 19-9 (5.2 U/mL), cytokeratin 19 fragment (2.2 ng/mL), and pro-gastrin-releasing peptide (66.9 pg/mL), were all within normal levels. The pulmonary tumor was highly suspected to be a metastasis from the colon cancer because of the tumor growth. No other metastases were observed on contrast-enhanced CT. We considered preoperative bronchoscopy. However, the patient preferred surgery for diagnostic and therapeutic purposes. Preoperative three-dimensional CT (3D-CT) angiography revealed that A7a branched from the right main PA, whereas A7b branched from the A8+9+10 as usual (Fig. 2a, b). The A7a and A7b were located on the ventral and dorsal sides of the basal vein, respectively.

**Fig. 3:**
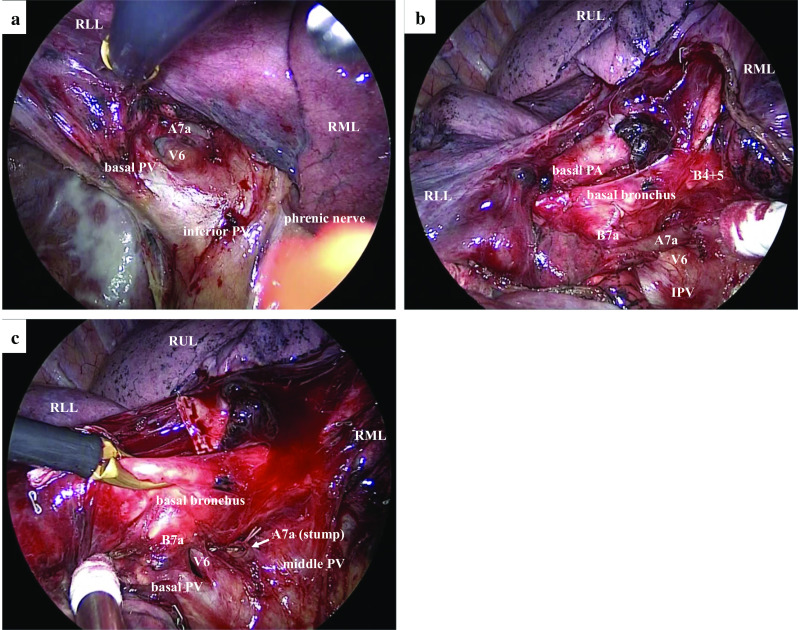
Intraoperative findings. **a** An aberrant mediastinal A7a on the cranial side of V6. **b** After division of the interlobar lung parenchyma between RML and RLL. **c** After dissection of the aberrant mediastinal A7a. *PV* pulmonary vein; *RLL* right lower lobe; *RML* right middle lobe; *RUL* right upper lobe

Right basal segmentectomy was performed via video-assisted thoracic surgery. The interlobar fissure between the middle and lower lobes was incomplete. Then, we dissected around the inferior pulmonary vein (IPV) to identify the border of the middle and lower lobes. Subsequently, during dissection of the cranial side of V6, we found the A7a that was close to V6 (Fig. 3a). The A7a branching from the right main PA was observed behind the V4+5 and middle lobe bronchus during surgery (Fig. 3b). The basal PA except for A7a was divided using a stapler, and A7a was ligated and divided (Fig. 3c). Then, the basal bronchus was divided using the stapler. After the vein of the basal segment (V7–10) was divided, the intersegmental plane was dissected using the stapler along the inflation and deflation lines. Intraoperative frozen section diagnosis revealed that the tumor was compatible with a metastasis from the colon cancer. The total operation time was 144 min, and the total blood loss volume was 30 mL.

The postoperative course was uneventful. The tumor had reached a maximum diameter of 20 mm, and the final pathologic diagnosis was combined small cell carcinoma and adenocarcinoma as opposed to the frozen section diagnosis. The pathological staging of the tumor was p-T1cN0M0, p-stage IA3. Although we proposed completion right lower lobectomy with systemic mediastinal lymph node dissection, the patient did not agree. The patient received adjuvant chemotherapy (XELOX) for colon cancer after a discussion with the cancer board in our institution. There was no recurrence of lung and colon cancer at a 1-year follow-up.

## Discussion

Although a previous report showed various patterns of branches of PA and bronchus in the right lung using 3D-CT, no reports have revealed that only A7a branched from the right main PA [3]. Only two case reports identified lung resections with A7 branching from the right main PA [1, 2]. One report showed an aberrant mediastinal inferior lobar branch [A6+ common basal artery (A7–10)] originating from the right main PA, which was located between the superior pulmonary vein (SPV) and intermediate bronchus similar to our case [1]. In our case, the size of the mediastinal A7a was smaller than that of the PA observed in the previous case. Another report also revealed A7 branching from the right main PA in a patient who underwent right middle lobectomy for lung cancer [2]. In this case, all A7 branched from the main PA, whereas in our case only A7a branched from the right main PA and A7b branched as usual from the A8+9+10. Careful attention is necessary to avoid damaging the mediastinal A7 branch especially during dividing the interlobar fissure of the middle and lower lobes.

According to a previous report, the branching pattern of B7 and A7 was classified into four types: B^7^a type, B^7^ab type, B^7^b type, and BX^7^ type. The B^7^a was the most common (74.8%) among the other types: B^7^ab type (14.8%), B^7^b type (4.8%), and BX^7^ type (5.6%) [3]. In our case, the PA pattern and bronchus branching were compatible with the B^7^ab type, in which there are two bronchi (B7a and B7b) and two arteries (A7a and A7b). However, no report has shown only A7a branching from the right main PA in the B^7^ab type. We labeled the A7a branching type as “aberrant mediastinal A7a.”

Thoracic surgeons should consider the possibility of an aberrant mediastinal A7a branching during anatomical resection of the middle or the lower lobe. In our case, we confirmed that the aberrant mediastinal A7a was surrounded by the basal bronchus and V6 as detected using 3D-CT angiography, which could help to avoid injury to this arterial branch. It is difficult to identify the A7a located deep from the interlobar surface when the interlobar fissure between the middle and lower lobes is incomplete. It is important to identify the A7a between the SPV and IPV before dividing the interlobar fissure between the middle and lower lobes by a stapling device. One of the most important points is to find the abnormal branching of the PA using 3D-CT at a preoperative conference or briefing.

## Conclusion

We herein report the first case of aberrant mediastinal A7a branching from the right main PA in a patient who underwent thoracoscopic basal segmentectomy for lung cancer. Thoracic surgeons should note the possibility of damage to the A7a during inadvertent tunneling or dividing of the interlobar lung parenchyma between the right middle and lower lobes. Three-dimensional computed tomography should be used effectively to obtain accurate information about anatomical variations of PA for safe anatomical pulmonary resection.

## Data Availability

Not applicable.

## Abbreviations

PA: Pulmonary artery
S9: Laterobasal segment
3D-CT: Three-dimensional computed tomography
A7: Medial basal segmental pulmonary artery
IPV: Inferior pulmonary vein
SPV: Superior pulmonary vein
